# Vitreoretinal Lymphoma: From Pathobiology to Integrated Diagnosis

**DOI:** 10.3390/cancers18203262

**Published:** 2026-10-10

**Authors:** Maria Carmela Saturno, Danilo Iannetta, Alessandro Lambiase, Marc D. de Smet

**Affiliations:** 1 Department of Sense Organs, Sapienza University of Rome, 00161 Rome, Italy; danilo.iannetta@uniroma1.it (D.I.); alessandro.lambiase@uniroma1.it (A.L.);; 2 Helvetia Retina Associates, MIOS SA, 1005 Lausanne, Switzerland; 3 New York Eye and Ear Infirmary of Mt Sinai, Icahn School of Medicine, New York, NY 10003, USA

**Keywords:** vitreoretinal lymphoma, multimodal imaging, IL-10, MYD88, liquid biopsy, molecular diagnostics

## Abstract

Vitreoretinal lymphoma (VRL) is a rare, high-grade intraocular malignancy able to mimic intermediate and/or posterior uveitis. Diagnosis is difficult because lymphomatous cells in ocular samples are scarce, fragile and rapidly degraded, particularly after corticosteroid exposure. This review examines how current knowledge of VRL pathobiology is reflected in its clinical, imaging and laboratory features and how these can inform diagnosis. Cytology remains the confirmatory test, while multimodal imaging helps localise vitreous, intraretinal and sub retinal pigmented epithelium (RPE) involvement and guides ocular sampling. Diagnostic yield depends on appropriate sample collection, handling and timely processing. Cytokine and molecular testing, including interleukin-10 (IL-10)/interleukin-6 (IL-6) assessment and detection of recurrent lymphoma-associated alterations such as MYD88 and CD79B, can provide diagnostic support when morphology is inconclusive. We also examine newer approaches designed to increase the information obtained from small-volume ocular samples through quantitative imaging and high-dimensional molecular profiling. The diagnostic value of each test depends on the anatomical distribution of disease and the characteristics of the specimen and is greatest when complementary findings are interpreted together.

## Simple Summary

Vitreoretinal lymphoma (VRL) is a rare intraocular malignancy able to mimic intermediate and/or posterior uveitis, making its diagnosis difficult. Identifying lymphomatous cells confirms the presence of a malignancy, although sampling and specimen processing can affect diagnostic yield. This review aims to examine how current knowledge of VRL pathobiology is reflected in its clinical, imaging and laboratory features and how these can be interpreted diagnostically. Moreover, it focuses on how disease distribution and specimen characteristics should guide the integration of complementary diagnostic evidence. Emerging approaches, designed to extract information from limited ocular material, are discussed as potential steps towards a more reliable diagnosis of VRL.

## 1. Introduction

Vitreoretinal lymphoma (VRL) is a rare, high-grade non-Hodgkin malignancy involving the vitreous, retina and retinal pigment epithelium (RPE); optic nerve and anterior segment localization is less common [1,2,3,4]. Population-based data are limited. In a 20-year series from British Columbia, the annual incidence was estimated at 0.047 cases per 100,000 persons per year, corresponding to approximately 0.47 cases per million population, with an apparent increase over time that may partly reflect improved diagnosis [5]. More than 95% of cases are diffuse large B-cell lymphomas (DLBCLs), whereas the remaining cases comprise rare T-cell and natural killer-cell subtypes [1,2,3,4].

In the fifth edition of the World Health Organization Classification of Haematolymphoid Tumours, primary large B-cell lymphoma of immune-privileged sites (IP-LBCL) includes primary large B-cell lymphomas arising in the central nervous system, vitreoretina and testis [6]. This classification refers specifically to primary lymphomas arising at these immune-privileged sites and does not encompass secondary vitreoretinal involvement. VRL and PCNSL are closely related biologically [1,3]. Primary VRL (PVRL) refers to disease arising primarily within the vitreoretinal compartment, whereas secondary VRL denotes vitreoretinal involvement occurring in association with PCNSL or, less commonly, systemic lymphoma [1,2,3,4]. Rare T-cell and NK-cell VRLs are therefore outside the WHO IP-LBCL category.

Ocular disease may precede, accompany or follow CNS lymphoma. CNS involvement develops in 56–90% of patients presenting with PVRL, commonly within 16–24 months, whereas ocular involvement occurs in 5–25% of patients with PCNSL [1,2,3]. CNS progression is the principal determinant of survival, with 65–85% of patients dying from CNS lymphoma [1,4,7].

Diagnosis is often delayed because VRL may mimic intermediate and/or posterior uveitis. Blurred vision, floaters, vitreous cells and retinal or subretinal infiltrates are nonspecific, and corticosteroid treatment may produce transient improvement [1,2,3,4,8]. Cytological demonstration of lymphomatous cells is confirmatory, but its sensitivity is limited by low tumour cellularity, rapid cellular degeneration, prior corticosteroid exposure and pre-analytical factors related to specimen collection and processing [2,3,4,5,6,7,8,9]. Reported sensitivities range from 31% to 87.5%, and negative cytology does not exclude VRL [4,8].

As the biology of VRL has become better defined, the diagnostic approach has expanded beyond cytology to incorporate clinical assessment, multimodal imaging, immunophenotyping, cytokine analysis and molecular testing [1,2,3,4,8]. These methods provide complementary information, particularly when cytology is inconclusive.

Reducing diagnostic uncertainty is clinically relevant because delayed recognition may postpone appropriate CNS staging and disease-specific treatment in a lymphoma whose prognosis is strongly influenced by CNS involvement.

This review focuses on how VRL pathobiology informs clinical, imaging and laboratory diagnosis, the limitations of established methods and the role of emerging approaches.

## 2. Methodology

This narrative review was based on a targeted literature search focused on the pathobiology and diagnosis of vitreoretinal lymphoma. PubMed/MEDLINE and Scopus were searched from inception to July 2026 using combinations of terms related to “vitreoretinal lymphoma”, “primary vitreoretinal lymphoma”, “intraocular lymphoma”, “diagnosis”, “vitreous biopsy”, “cytology”, “flow cytometry”, “interleukin-10”, “MYD88”, “molecular diagnostics”, “multimodal imaging”, “optical coherence tomography”, “liquid biopsy”, “next-generation sequencing” and “machine learning”. Reference lists of relevant original studies, consensus statements, systematic reviews and meta-analyses were also examined to identify additional publications. Priority was given to studies directly addressing VRL pathobiology, diagnostic performance, specimen handling and emerging diagnostic approaches.

## 3. Pathobiology

B-cell VRL predominantly shows an Activated B-Cell-like (ABC) phenotype of DLBCL, sharing molecular features with PCNSL [3,4,10]. In PVRL, the presence of highly somatically mutated immunoglobulin heavy chain (IGH) genes is consistent with a post-germinal-centre B-cell origin. Immunogenetic analyses have demonstrated a restricted immunoglobulin gene repertoire, characterised by frequent IGHV4-34 usage and, in a subset of cases, stereotyped IGHV3-7 rearrangements. This biased repertoire supports the involvement of antigen-driven selection in PVRL lymphomagenesis [10,11,12,13]. Genomic profiling has further demonstrated that VRL shares molecular features of the MYD88/CD79B-mutated (MCD/cluster 5) molecular subtype of ABC-DLBCL, a subgroup enriched in lymphomas involving immune-privileged sites. Recurrent alterations include mutations in MYD88, CD79B, PIM1, IGLL5, BTG1/2, TBL1XR1 and ETV6, together with frequent 9p21/CDKN2A deletions [3,4,10,11,12,13,14].

Bonzheim et al. identified MYD88 alterations in 74% of VRL cases, almost all involving the recurrent p.L265P hotspot, and CD79B mutations in 55% [11]. MYD88 L265P activates MYD88-IRAK signalling with downstream NF-κB and JAK-STAT3 activation, whereas CD79B mutations promote chronic active B-cell receptor signalling and NF-κB activation [14,15]. Their frequent co-occurrence in VRL is consistent with the MCD/cluster 5 molecular profile of ABC-DLBCL [11,14,15]. Functional evidence for these pathways, however, derives largely from studies of ABC-DLBCL rather than VRL-specific models [14,15].

To date, the anatomical origin of the VRL cells is uncertain. Two models have been proposed: local malignant transformation of B cells recruited to the eye and ocular homing of a pre-existing malignant clone [1,4,16,17].

In the first model, chronic antigenic and/or inflammatory stimulation within the immune-privileged ocular microenvironment may promote B-cell recruitment and survival, creating conditions in which the acquisition of lymphoma-associated genetic alterations could promote malignant transformation.

An infectious contribution to this process has been considered. In a molecular analysis of VRL specimens, Human herpesvirus 8 (HHV-8) DNA was detected in six of 32 cases (18.8%), Epstein–Barr virus (EBV) DNA in two of 21 (9.5%) and Toxoplasma gondii (Tg) DNA in two of 16 (12.5%) [18]. Tg DNA was also identified in micro-dissected lymphomatous cells, but not normal cells, in two of 10 cases of VRL [19]. These findings support the possibility that antigenic stimulation contributes to lymphomagenesis in a subset of patients.

EBV has been implicated in the setting of impaired immune surveillance [20,21]. HIV-associated PCNSL is strongly associated with EBV infection, and loss of EBV-specific T-cell control in advanced HIV infection may allow expansion of EBV-infected B cells [20]. Nevertheless, EBV-associated VRL is not confined to overtly immunocompromised patients. Zafar et al. described EBV-positive VRL in two elderly patients without systemic immunodeficiency and proposed that age-related immunosenescence could favour EBV-driven lymphoproliferation. These cases showed CD30 expression and lacked the high-frequency MYD88 and CD79B alterations, suggesting that EBV-associated VRL may be biologically distinct. The authors also proposed that impaired local immune surveillance in chronically inflamed eyes may favour expansion of EBV-transformed B cells [21].

Further evidence came from Liu et al., who analysed vitreous samples from 53 patients with VRL, all HIV-negative and without known immunodeficiency [22]. EBV DNA was detected by PCR in 15 patients (28%). In three specimens with high viral copy numbers and sufficient material for immunofluorescence, Epstein–Barr nuclear antigen 1 (EBNA1) colocalized with CD20-positive malignant cells, confirming the presence of EBV within the neoplastic B-cell population in those samples. EBV-positive cases had higher vitreous IL-10 concentrations (*p* = 0.006) and shorter progression-free survival (*p* = 0.004) than EBV-negative cases [22].

Experimental models have explored routes of lymphomatous cell dissemination between systemic, CNS and ocular compartments, with the earliest studies using a T-cell lymphoma model. Assaf et al. showed that intraperitoneally inoculated Rev-2-T-6 lymphomatous T-cells preferentially involved the eye and CNS in young syngeneic BALB/c mice [23]. Histopathological mapping indicated that lymphomatous cells entered the CNS mainly through the choroidal plexus and cranial nerves, spread within the brain and then migrated along the optic nerve sheath towards the eye. Orbital infiltration occurred independently of this brain–optic nerve route, while no spontaneous retrograde spread from the eye to the brain was observed [24].

Intravitreal models further characterised lymphoma behaviour within the eye. Following intravitreal inoculation of Rev-2-T-6 cells into immunocompetent BALB/c mice, lymphomatous cells migrated through the retina and accumulated in the subretinal space within 2–4 weeks. Breaching of the RPE with extension into the choroid and sclera was rare and occurred only after more than 2 months, supporting a barrier function of the RPE [25]. In contrast, systemic LFA-1/CD11a blockade allowed spread along the optic nerve sheath to the brain and contralateral eye. A similar pattern occurred in severe combined immunodeficient mice, indicating a role for LFA-1-dependent immune surveillance in limiting extraocular spread [26]. Li et al. examined a B-cell lymphoma model using intravitreal inoculation of human CA46 cells into severe combined immunodeficient mice. Malignant cells initially localised along the retinal surface, infiltrated the retina, preferentially expanded in the subretinal space and rarely crossed the RPE into the choroid; CNS dissemination was also observed [27].

Human tissue studies suggest that ocular homing of lymphomatous cells is mediated by chemokine signalling. Chan et al. found high expression of the chemokine receptors CXCR4 and CXCR5 on lymphomatous B cells, whereas their respective ligands were expressed by the RPE, with prominent CXCL13 (BLC) expression and more limited CXCL12 (SDF-1) detection. The authors proposed that these RPE-derived chemokines may attract malignant B cells from the choroidal circulation and contribute to their sub-RPE localization [28]. Related pathways have also been described in PCNSL. Smith et al. detected CXCL13 in all 24 PCNSL specimens examined, together with CXCR5 expression by malignant B cells; although CXCL13 was also detected in the vascular endothelium, transcripts were confined to tumour cells, consistent with endothelial transcytosis rather than local synthesis [29]. Venetz et al. demonstrated vascular and perivascular CXCL12 expression and CXCR4 expression by malignant B cells in PCNSL. In vitro, CXCL9 enhanced CXCL12-induced, CXCR4-dependent migration, providing a potential mechanism for the characteristic perivascular localization of lymphomatous cells in PCNSL [30].

Within the vitreoretinal compartment, malignant cells reside in an immune-privileged microenvironment that can favour their persistence. The inner and outer blood–retinal barriers, formed by tight junctions between retinal vascular endothelial cells and RPE cells, restrict paracellular diffusion from the vascular and perivascular compartments into the retina. Local immunoregulatory mechanisms involving TGF-β2, Fas ligand, PD-L1 and other soluble and membrane-bound mediators limit inflammatory and cytotoxic immune responses [31,32]. Experimental models have also shown that ocular immune privilege can promote the survival of intraocular neoplastic cells by restraining both adaptive and innate immune mechanisms [31].

Interleukin-10 (IL-10) is a key immunoregulatory cytokine in the VRL microenvironment. Chan et al. detected abundant IL-10 mRNA in micro-dissected lymphomatous cells from all six VRL cases examined, providing direct evidence that malignant B cells contribute to intraocular IL-10 production [18]. Beyond its B-cell growth-promoting activity, IL-10 suppresses antitumor immunity by reducing MHC class II and co-stimulatory molecule expression, limiting antigen presentation and effector T-cell responses, facilitating tumour immune escape [1,33,34]. In ABC/MCD-DLBCL, IL-10 also acts directly on lymphomatous cells through an autocrine IL-10/IL-10R axis, activating JAK1-STAT3 signalling, promoting cell survival and increasing PD-L1 expression [33,35]. The effects of tumour-derived IL-10 are context-dependent. In an immunocompetent ABC-DLBCL model, selective loss of tumour-derived IL-10 resulted in a more T-cell-exhausted and angiogenic microenvironment, showing that its effects depend on the cellular and immune context [35].

The immune profile of VRL extends beyond IL-10. Usui et al. analysed 38 mediators in vitreous samples from 28 eyes with B-cell VRL and found significantly increased levels of 17 mediators compared with 27 uveitis controls. These included CXCL13, CXCL12, CCL2, CCL3, CCL4, CCL5, CXCL9, CXCL10, IL-6, IL-8, IL-10, IFN-γ, oncostatin M, FGF2, Fas ligand and granzymes A and B. Correlations between several of these mediators indicated coordinated activity across pathways involved in B-cell growth and trafficking, inflammatory-cell recruitment and cytotoxic responses [34].

## 4. Clinical Features

**Figure 1:**
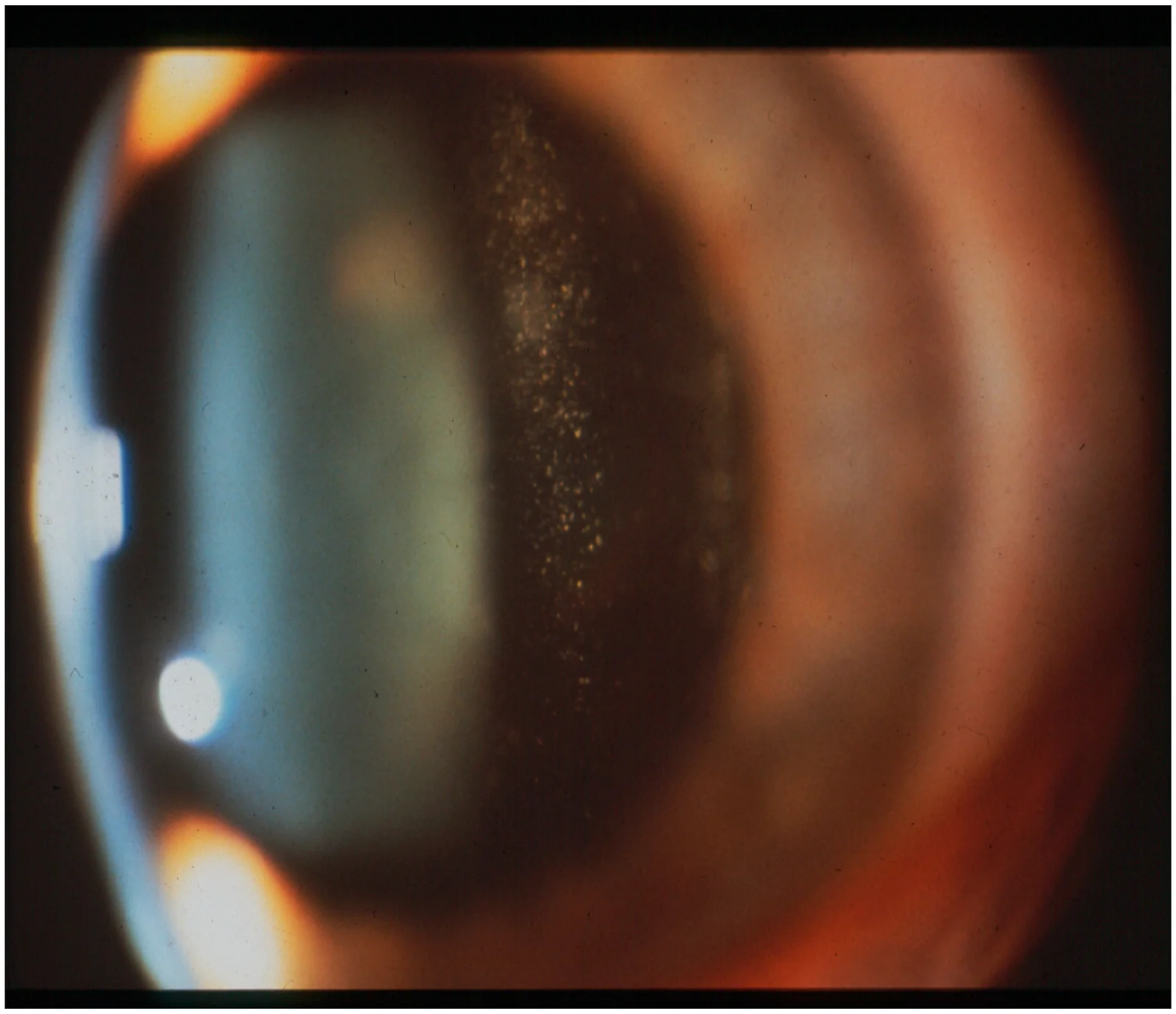
Anterior vitreous cellularity in vitreoretinal lymphoma. Slit-lamp photograph showing marked anterior vitreous cellular infiltration in a patient with vitreoretinal lymphoma, clinically mimicking inflammatory vitritis. Original image from MIOS SA, Lausanne, Switzerland.

VRL is a classical masquerade syndrome that, mimicking intermediate and/or posterior uveitis, reports diagnostic delays from 6 to 40 months [1,2,3]. Patients are typically older adults presenting with floaters, blurred or decreased vision [2,16]. Bilateral disease occurs in 64–83% of cases and can be asymmetric. Anterior segment involvement is generally mild and may include trace anterior chamber cells and keratic precipitates, whereas pseudohypopyon is rare [1,2,3] (Figure 1).

Vitritis is one of the most common clinical manifestations and may range from mild to dense, with vitreous cells forming strands, clumps, sheets or veils along the vitreous fibrils, sometimes producing the characteristic “aurora borealis” appearance [1,2,36,37,38,39]. Preserved visual acuity despite substantial vitreous infiltration and little or no cystoid macular oedema is a characteristic clinical clue.

**Figure 2:**
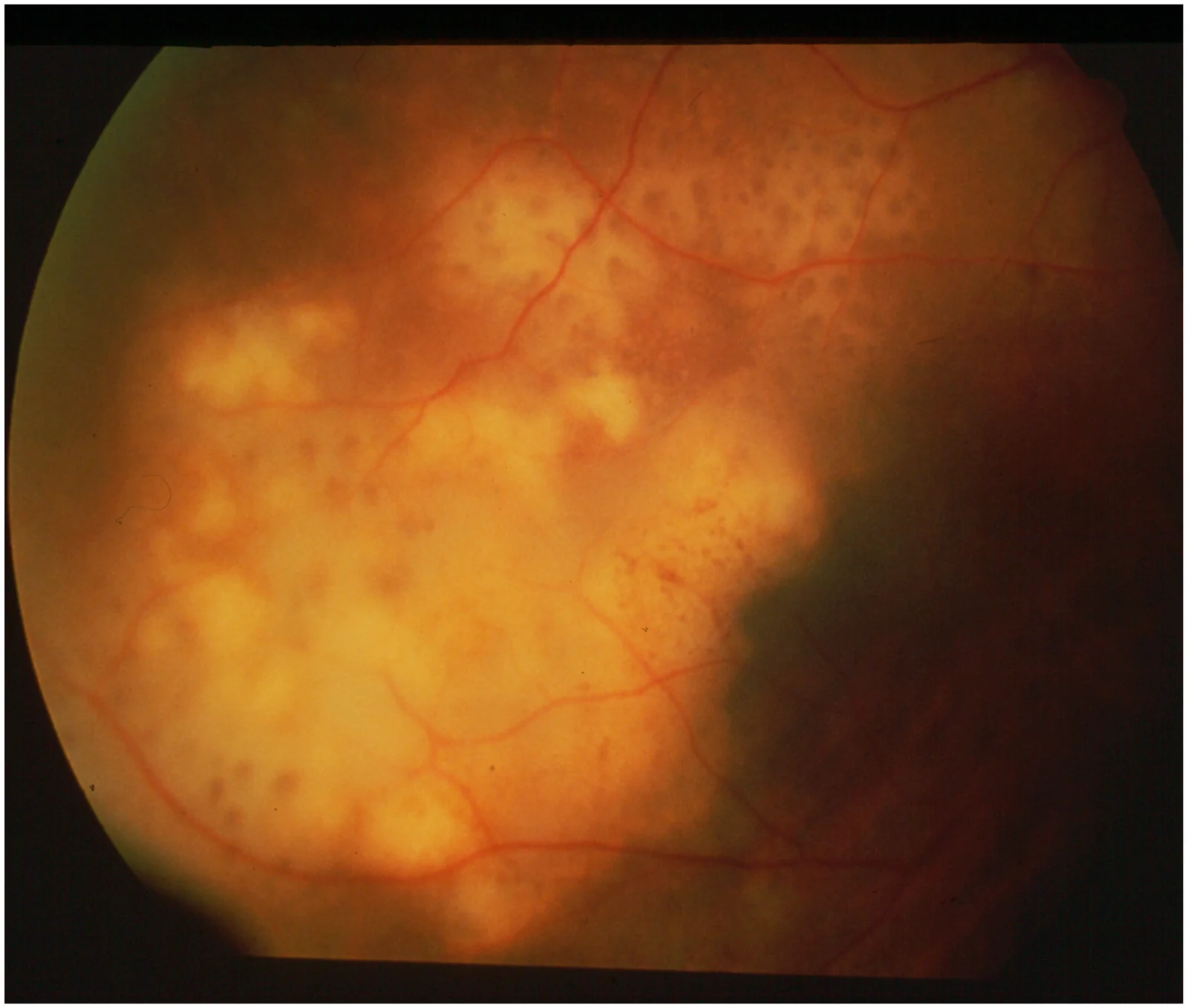
Subretinal infiltrates in vitreoretinal lymphoma. Fundus photograph showing multifocal creamy yellow-white subretinal infiltrates, with a mottled “leopard-skin” appearance. Original image from MIOS SA, Lausanne, Switzerland.

Retinal involvement may include multifocal creamy or yellow-white infiltrates located in the outer retina and/or RPE, which may coalesce and produce a mottled or “leopard-skin” appearance. Additional manifestations include punctate deep retinal lesions, RPE alterations, subretinal infiltrates, exudative retinal detachment, and, following regression, RPE atrophy or subretinal fibrosis. Retinal haemorrhage, retinal vasculitis, macular oedema and necrotizing retinitis are uncommon at presentation but may occur in advanced or atypical disease. Optic nerve involvement may also occur [1,2,3,4,36,37,38,39] (Figure 2). Corticosteroids can transiently reduce ocular inflammation and lymphomatous cell burden, further delaying diagnosis; recurrence or progression during corticosteroid tapering is therefore an important diagnostic clue [2,16]. The principal clinical features reported across consensus recommendations and clinical series are summarised in Table 1 [1,2,3,4,36,37,38,39].

## 5. Multimodal Imaging

Multimodal imaging helps identify typical patterns, define the anatomical distribution of VRL and monitor structural changes over time. It does not establish the diagnosis on its own and should be interpreted together with laboratory findings [1,2,40].

Colour fundus photography may reveal vitreous haze and cellular aggregates, retinal or subretinal lesions, RPE mottling and perivascular sheathing. In a series of 43 eyes from 23 patients, vitritis and subretinal lesions were each present in 46% of eyes, while optic disc oedema and exudative retinal detachment were seen in 10% and 12% of eyes, respectively. Ultra-widefield (UWF) imaging disclosed additional findings beyond the conventional 30° field in 47% of eyes, highlighting the extent of peripheral involvement [41].

**Figure 3:**
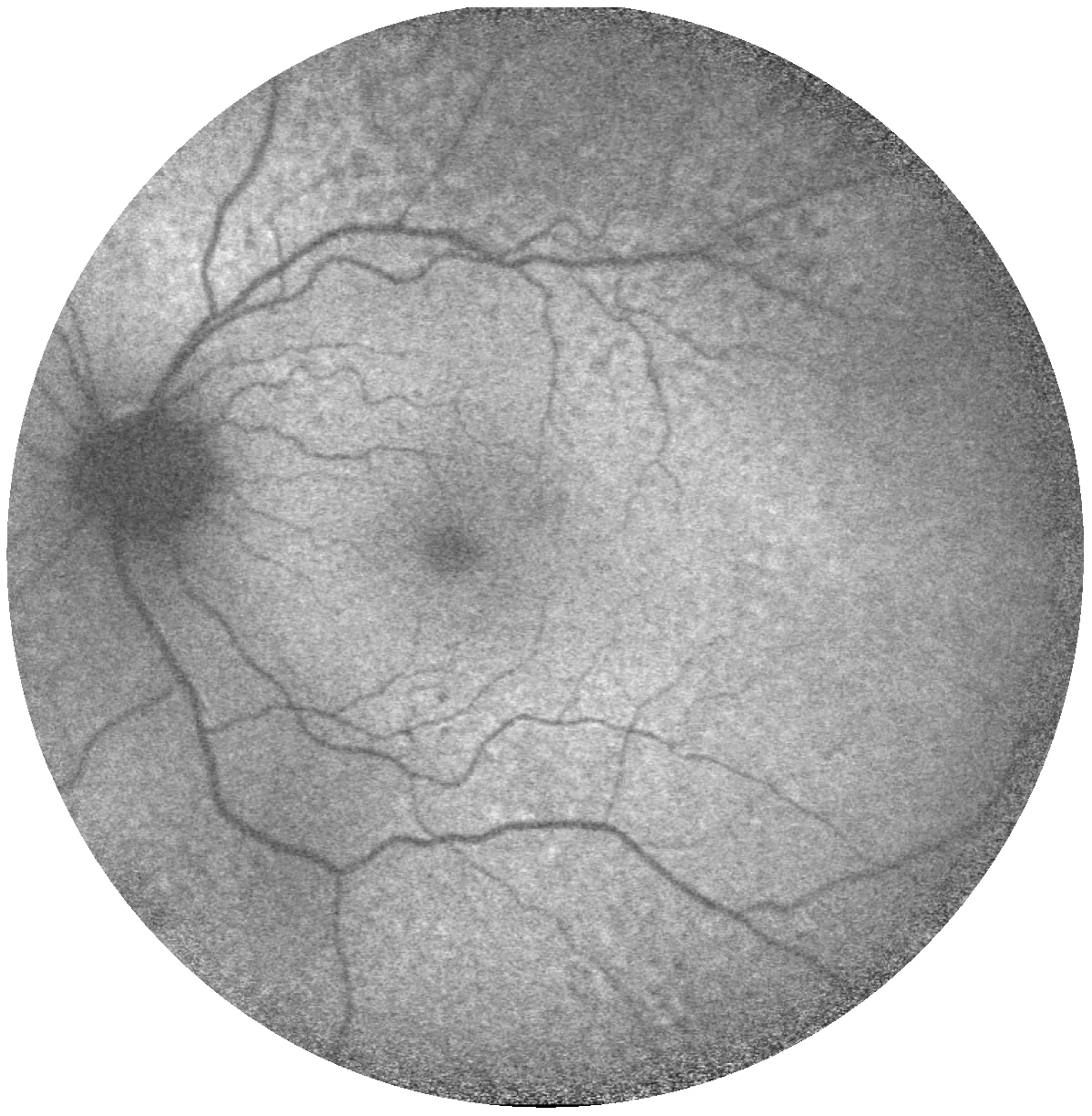
Fundus autofluorescence in vitreoretinal lymphoma. FAF image showing a granular pattern of mixed hyper- and hypoautofluorescent foci, consistent with RPE alterations in vitreoretinal lymphoma. Original image from MIOS SA, Lausanne, Switzerland.

Fundus autofluorescence (FAF) provides a non-invasive assessment of RPE alterations associated with VRL. The characteristic finding is a granular or stippled pattern of hyper- and hypoautofluorescent foci (Figure 3). Hyperautofluorescence may reflect RPE dysfunction associated with sub-RPE lymphomatous infiltration, whereas hypoautofluorescence may result from masking of the normal RPE signal by overlying infiltrates or from RPE atrophy following therapy [40,42,43,44]. Different autofluorescence patterns may coexist within the same area and change with disease evolution and treatment. FAF alone cannot distinguish active from inactive disease and a normal FAF does not exclude VRL. Casady et al. reported no remarkable FAF findings in seven of 18 eyes, including five eyes with active disease [44]. Conversely, hyperautofluorescent dots were also observed in an eye with inactive disease [40,44]. Across 191 eyes, a mixed hypo/hyperautofluorescent pattern was present in 65.99% (95% CI, 40.04–88.05%), whereas 32.17% showed a normal FAF, highlighting the limited sensitivity of this tool [42].

Spectrally resolved colour-coded FAF has been investigated as a complementary approach to conventional blue and green FAF. In a retrospective study of 20 eyes from 10 patients, the characteristic granular pattern was detected equally by all three modalities (13/13 eyes). Colour-coded FAF appeared to better delineate retinitis-like lesions in 2/2 eyes, compared with 0/2 on blue and green FAF, and lymphomatous RPE detachments in 4/4 eyes, compared with 1/4 using either conventional modality; it also depicted RPE atrophy, sub-RPE deposits and perivascular infiltrates [43].

**Figure 4:**
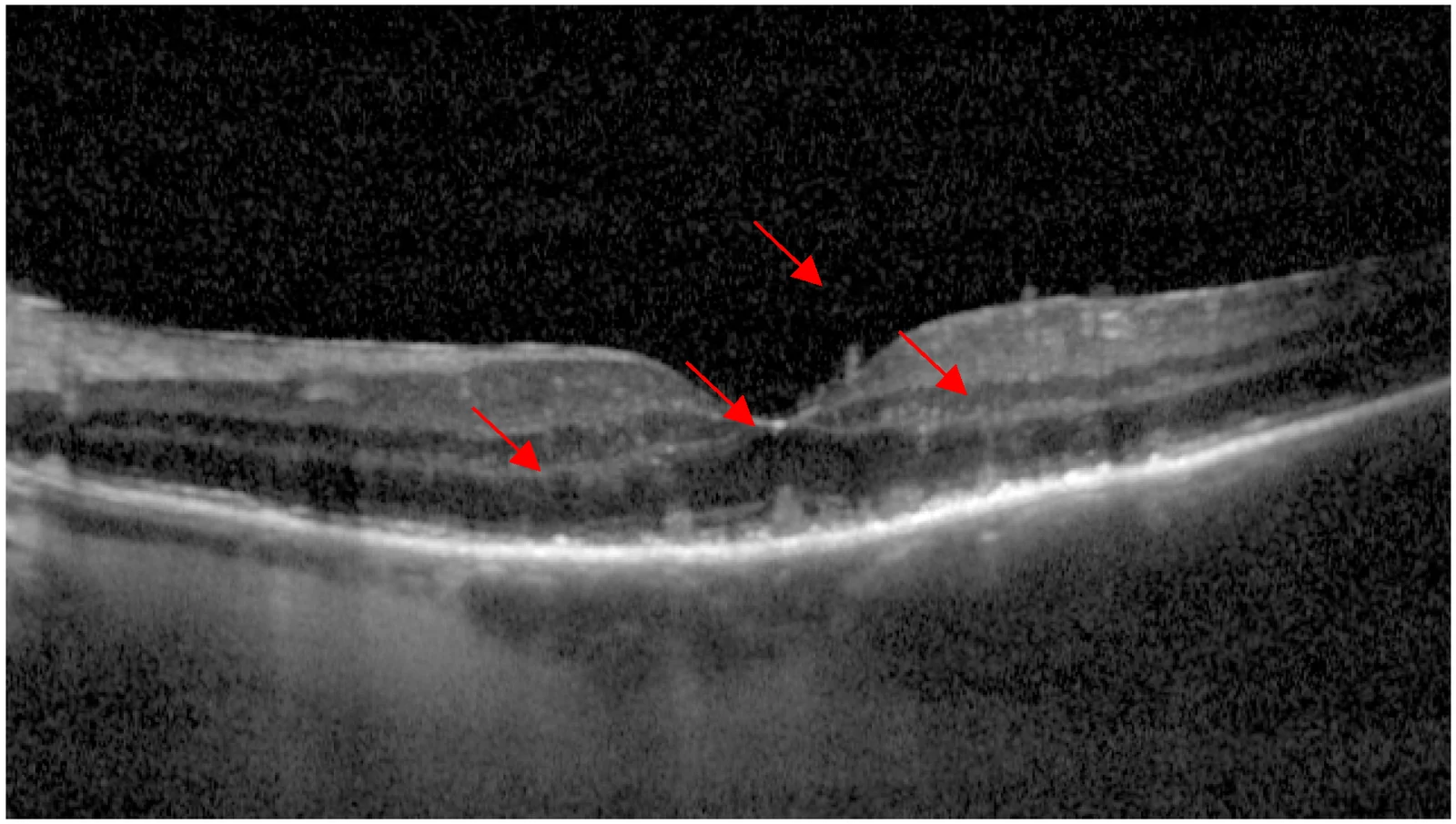
Optical coherence tomography features of vitreoretinal lymphoma. Optical coherence tomography showing hyper-reflective abnormalities involving the preretinal, intraretinal, subretinal and sub-RPE compartments (arrows). Original image from MIOS SA, Lausanne, Switzerland.

Optical coherence tomography (OCT) allows for detailed assessment of VRL involvement in the posterior vitreous, retina, subretinal space and sub-RPE compartment (Figure 4) [1,2,3,4,42,43,44,45,46,47,48,49,50]. In a multicentre series of 182 eyes from 115 patients, VRL-associated hyper-reflective abnormalities were identified in the sub-RPE compartment in 91% of eyes, the subretinal space in 43% and the intraretinal compartment in 7%. Isolated sub-RPE involvement was the most frequent pattern (55%), followed by combined sub-RPE and subretinal involvement (29%); isolated subretinal lesions occurred in 9%, whereas isolated intraretinal involvement was rare (0.5%) [45]. The morphology of OCT findings varies according to the involved compartment. Sub-RPE infiltration may appear as thickening of the RPE–Bruch’s membrane complex, a shallow hyper-reflective pigment epithelial detachment (PED) or a larger dome-shaped PED. Subretinal infiltration may form focal rounded deposits or broad band-like hyper-reflective lesions, whereas intraretinal infiltration may extend from the outer nuclear layer towards the nerve fibre layer [45]. Across 591 eyes, pooled prevalences were 45.6% for RPE hyper-reflective material, 43.5% for RPE–Bruch’s membrane infiltration, 39.7% for subretinal hyper-reflective material, 39.3% for fuzzy outer-retinal changes and 15.3% for PED [42].

A distinct pattern of intraretinal involvement is represented by vertical hyper-reflective lesions (VHRLs). Deák et al. identified VHRLs in seven of 12 eyes (58.3%); these lesions extended through the neuroretina, were frequently localised around second- and third-order retinal vessels, often preceded the development of sub-RPE deposits and, in most cases, resolved with minimal or no scarring following therapy. Their perivascular distribution led the authors to hypothesise that VHRLs may represent early infiltrates of lymphomatous cells originating from retinal vessels and extending towards the sub-RPE compartment [46].

Preretinal deposits are an additional OCT finding, ranging from small superficial hyper-reflective foci to larger proliferations projecting into the vitreous [45,49]. A “stalagmite-like” configuration of larger preretinal lesions has been described as a suggestive but non-specific pattern; a similar morphology may also occur in fungal endophthalmitis [49].

Chen et al. evaluated 158 eyes from 81 patients with VRL and identified RPE disruption in 51 eyes, with 47 of these already present at the initial visit. All RPE disruption lesions occurred in eyes with PED and over areas of sub-RPE infiltration, and no Bruch’s membrane disruption was observed. Major disruptions (>300 µm) were more frequently associated with subretinal infiltration, whereas minor disruptions (<300 µm) were associated with intraretinal infiltration [50]. The authors proposed that RPE disruption may represent a potential pathway for lymphomatous cell extension between the sub-RPE, subretinal and intraretinal compartments. OCT also provides longitudinal assessment of structural response to therapy. In the RPE-disruption cohort, the median time to resolution was 2 months for RPE disruption and 3 months for PED. Eyes with minor disruption had significantly better final visual acuity, with significant improvement from baseline observed in the minor-disruption group [50]. Persistent OCT abnormalities may remain for more than 6 months after treatment and do not necessarily indicate active disease [42]. The basis of this persistence remains unclear.

No single OCT feature combines high sensitivity and specificity for VRL. Guan et al. compared 45 eyes with VRL with 40 eyes affected by acute syphilitic posterior placoid chorioretinitis, chronic sympathetic ophthalmia or idiopathic multifocal choroiditis. Vitreous cells showed high sensitivity (93%) but low specificity (33%). Intraretinal infiltration excluding the incomplete vertical hyper-reflective form and confluent RPE detachments showed 100% specificity within this comparator cohort; preretinal deposits and band-like subretinal infiltration showed specificities of 92.5% and 90%, respectively, but sensitivities below 50% [47].

El Zein et al. compared 95 VRL eyes with 86 eyes affected by intermediate uveitis or sarcoid posterior uveitis [48]. Preretinal deposits (31.6% vs. 9.3%), intraretinal infiltrates (34.0% vs. 3.5%), inner-retinal hyper-reflective foci (15.8% vs. 0%), focal subretinal deposits (21.1% vs. 4.7%), RPE abnormalities (49.5% vs. 3.5%) and sub-RPE deposits (34.7% vs. 0%) were more frequent in VRL. Conversely, epiretinal membrane, central macular thickening, cystoid macular oedema and subretinal fluid occurred more frequently in uveitis. In multivariable models adjusted for age and sex, the absence of an epiretinal membrane and of central macular thickening remained independently associated with VRL [48].

En face OCT angiography (OCTA) may disclose perivascular intraretinal abnormalities. Chen et al. identified perivascular flower-bud-like lesions (PFBLs) on midretinal en face slabs in 12 of 35 eyes (34.3%) from eight of 23 patients with VRL; in 10 of the 12 affected eyes, PFBLs were detected within 6 months of symptom onset. On en face OCTA, PFBLs appeared as punctate points or confluent bands surrounding retinal vessels, with both arterial and venous involvement. On OCT B-scans, they appeared as hyper-reflective full-thickness intraretinal lesions and could colocalize with sub-RPE deposits or RPE irregularities. PFBLs could not always be identified on fundus photography or fluorescein angiography (FA). In all eyes with follow-up, PFBLs attenuated or resolved months after chemotherapy or diagnostic vitrectomy [51].

Retromode imaging has also been investigated. In a pilot series, seven eyes from five patients were suitable for analysis, with applicability limited by media opacity, particularly vitritis, which substantially degraded image quality. Using laterally deviated infrared illumination, Retromode generates pseudo-three-dimensional images of retinal topography and revealed additional or more extensive deep-retinal and RPE abnormalities than FAF. These findings also enabled targeted OCT acquisition in areas not routinely covered by macular scanning, where subretinal or sub-RPE infiltration was subsequently confirmed [52].

Quantitative assessment of the choroid using swept-source OCT/OCTA has also been investigated. Zhou et al. studied 44 eyes from 29 patients with VRL and found a significantly lower choroidal vascularity index (CVI) than in age- and sex-matched healthy controls across all analysed ETDRS regions, whereas baseline choroidal thickness (CT), choroidal vessel volume (CVV) and choroidal stromal volume (CSV) did not differ significantly. Choriocapillaris perfusion area was greater in the perifoveal and extrafoveal regions of VRL eyes [53]. During intravitreal methotrexate treatment, CT, CVV and CSV decreased significantly from the induction phase onwards, whereas CVI remained stable and choriocapillaris perfusion showed no consistent longitudinal change. The lower baseline CVI was not significantly different from controls after treatment. The authors considered these changes more consistent with reactive T-cell infiltration of the choroid than with direct tumour-cell infiltration [53].

**Figure 5:**
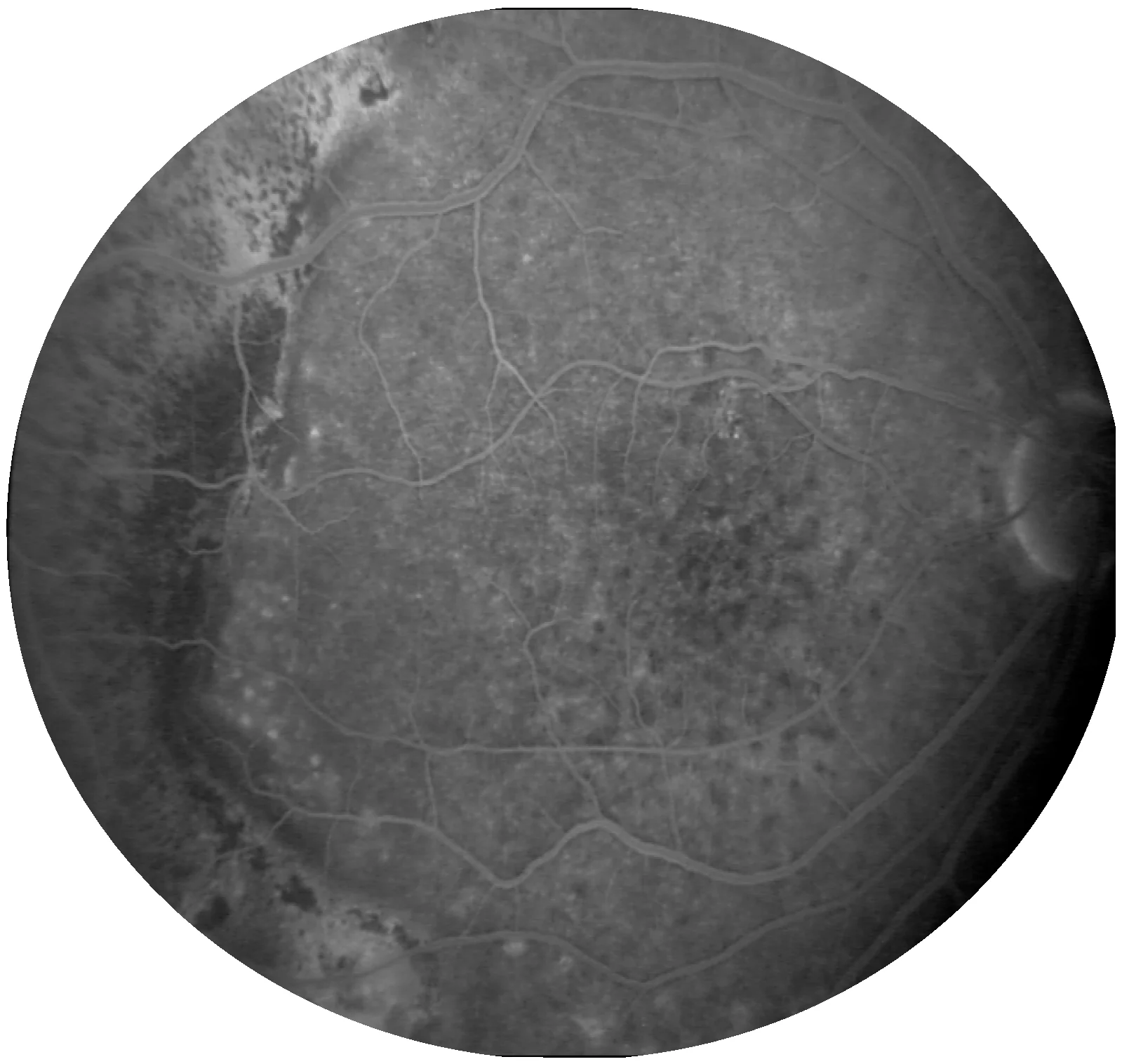
Fluorescein angiography in vitreoretinal lymphoma. Fluorescein angiography showing a mottled “leopard-spot” pattern with multiple small hypo- and hyperfluorescent foci, consistent with multifocal retinal and RPE involvement in vitreoretinal lymphoma. Original image from MIOS SA, Lausanne, Switzerland.

FA may show a characteristic mottled or “leopard-spot” pattern, consisting of multiple small hypo- and hyperfluorescent spots (Figure 5). A suggestive finding is the presence of clusters of well-defined, round hypofluorescent lesions, approximately 50–250 μm in diameter, which remain hypofluorescent from the early through the late phases because of persistent masking of the underlying choroidal fluorescence. These lesions correspond to punctate whitish fundus lesions and may involve both the posterior pole and the retinal periphery [2,54]. Leopard spots had a pooled prevalence of 51.2% (95% CI, 39.2–63.1%), while multiple hypofluorescent spots had a pooled prevalence of 40.6% (95% CI, 29.0–52.7%). Window defects and staining lesions had pooled prevalences of 40.8% and 33.9%, respectively [42]. Hypofluorescent spots on FA may correspond to hyperautofluorescent spots on FAF [44] and to nodular hyper-reflective abnormalities at the RPE level on OCT [44,54]. Angiographic inflammatory features are less characteristic: optic nerve head leakage, a vasculitic pattern and cystoid macular oedema were reported in 31.2%, 26.1% and 9.2% of eyes, respectively [42].

Indocyanine green angiography (ICGA) provides complementary information, although its findings have been less reported. Small hypofluorescent lesions may be visible in the early phase, are typically fewer than the corresponding hypofluorescent lesions on FA, and often become less conspicuous on late images [2,54]. Late persistence is uncommon, with vanishing and persistent hypofluorescent spots reported in 40.7% and 7.5% of eyes, respectively, although these estimates were based on only two studies [42].

B-scan ultrasonography may offer additional diagnostic information, particularly when media opacity limits fundus imaging. Gu et al. compared 106 eyes with biopsy-confirmed VRL and 86 eyes with uveitis and found that retinal thickening or occupying lesions (20% vs. 1%) and a centrifugal condensation pattern of vitreous haze (46% vs. 15%) were more frequent in VRL, whereas vitreoretinal adhesion was less common (6% vs. 23%) [55].

The main imaging features of VRL and their clinical relevance are summarised in Table 2.

## 6. Vitreous Biopsy and Specimen Handling

Diagnostic pars plana vitrectomy (PPV) is the preferred technique for vitreous sampling in suspected VRL [1,2,3,4]. The yield may be reduced by the scarcity and fragility of neoplastic cells, their rapid degeneration after collection, previous corticosteroid exposure and delays or inadequacies in specimen processing [2,56,57]. Lymphomatous cells may undergo morphological degradation within 60 min after removal from the eye, requiring prompt transport and processing [2].

The allocation of vitreous material should be planned with the pathologist before surgery, according to the intended analyses [56,57]. Systemic corticosteroids should be discontinued for at least 2 weeks before the procedure. An early undiluted vitreous sample may be obtained and both undiluted and diluted specimens should be analysed. If immediate processing is not feasible, specimen preservation should be agreed on in advance with the pathologist. A low vitrector cut rate, up to 1500 cuts/min, is recommended to minimise mechanical damage to lymphomatous cells. The optimal vitrectomy gauge remains undetermined, as no difference in cytological yield has been demonstrated between 20- and 25-gauge systems [2].

The extent of vitreous sampling may influence cellular yield. Mudhar et al. found that full PPV specimens contained 7.4- to 78-fold more cells than core vitreous biopsies, with a mean 31-fold increase in cellularity. In the two patients ultimately diagnosed with VRL, only the full PPV specimen provided diagnostic material [58].

Conventionally, 1–2 mL of undiluted vitreous is aspirated before fluid infusion through a syringe connected directly to the vitrectomy handpiece [2,56,59]. Mura et al. described a 25-gauge three-port technique using air as the sole infusion medium, allowing intraocular pressure to be maintained while avoiding infusion-fluid dilution and permitting collection of a larger volume of undiluted vitreous during PPV [59]. The vitreous sampling should preferentially select an area of high cellularity.

Diagnostic material can also be recovered from diluted vitreous and cassette fluid. Shiozaki et al. used a standardised protocol in which approximately 1 mL of undiluted vitreous was obtained before infusion, followed by submission of the entire vitrectomy cassette content for cytological analysis. Among 12 patients with VRL, Class IV or V cytology was obtained in 9/12 cases (75%) from the undiluted specimen alone and in 11/12 (92%) when cassette material was analysed. Although the difference was not statistically significant (*p* = 0.50), cassette analysis provided a positive result in two cases in which the initial specimen had been negative or suspicious [60].

Cell-block processing concentrates cells recovered from vitreous fluid into paraffin-embedded material, allowing morphological and immunohistochemical assessment. Kase et al. identified atypical lymphoid cells in 14/15 evaluable cell-block specimens (93.3%), compared with 5/14 conventional smears (35.7%); seven smear-negative samples were positive on cell block [61].

Ohe et al. evaluated 25-gauge vitrectomy under infusion, using targeted vitreous sampling for liquid-based cytology (LBC) and irrigating fluid for cell-block preparation. Atypical large lymphoid cells were detected in 26/29 LBC samples (89.7%) and 20/23 evaluable cell blocks (87.0%); the combined approach was positive in 28/29 VRL eyes (96.6%), with no characteristic cytological abnormalities in 77 control eyes. The only negative case had predominantly subretinal disease with minimal vitreous involvement [62].

Qin et al. developed a modified intraoperative collection system to reduce dilution of cassette fluid intended for genomic analysis. In 29 eyes with cytopathologically confirmed B-cell VRL, the modified protocol increased median DNA concentration from 1.2 to 14.5 ng/µL (*p* = 0.002) and improved sequencing success from 10/14 to 15/15 samples (*p* = 0.042) [63].

A negative vitreous cytology does not exclude VRL. When clinical suspicion remains high despite a nondiagnostic vitreous sample, repeat vitrectomy or, in selected cases, targeted subretinal or chorioretinal sampling may be required [2,56]. A chorioretinal biopsy is particularly relevant when lymphomatous involvement is predominantly retinal or sub-RPE, with little or no vitreous infiltration. More recent areas of infiltration should be preferentially targeted because lymphoma cells within older bulky lesions may already have degenerated; the inclusion of choroidal tissue may also complicate histological interpretation because of the accompanying reactive lymphocytic infiltrate [2]. In a retrospective series, chorioretinal biopsy provided a definitive diagnosis in 59% of cases and contributed to excluding VRL in a further 31%, although evidence remains limited [8].

## 7. Cytology, Immunocytochemistry and Flow Cytometry

**Figure 6:**
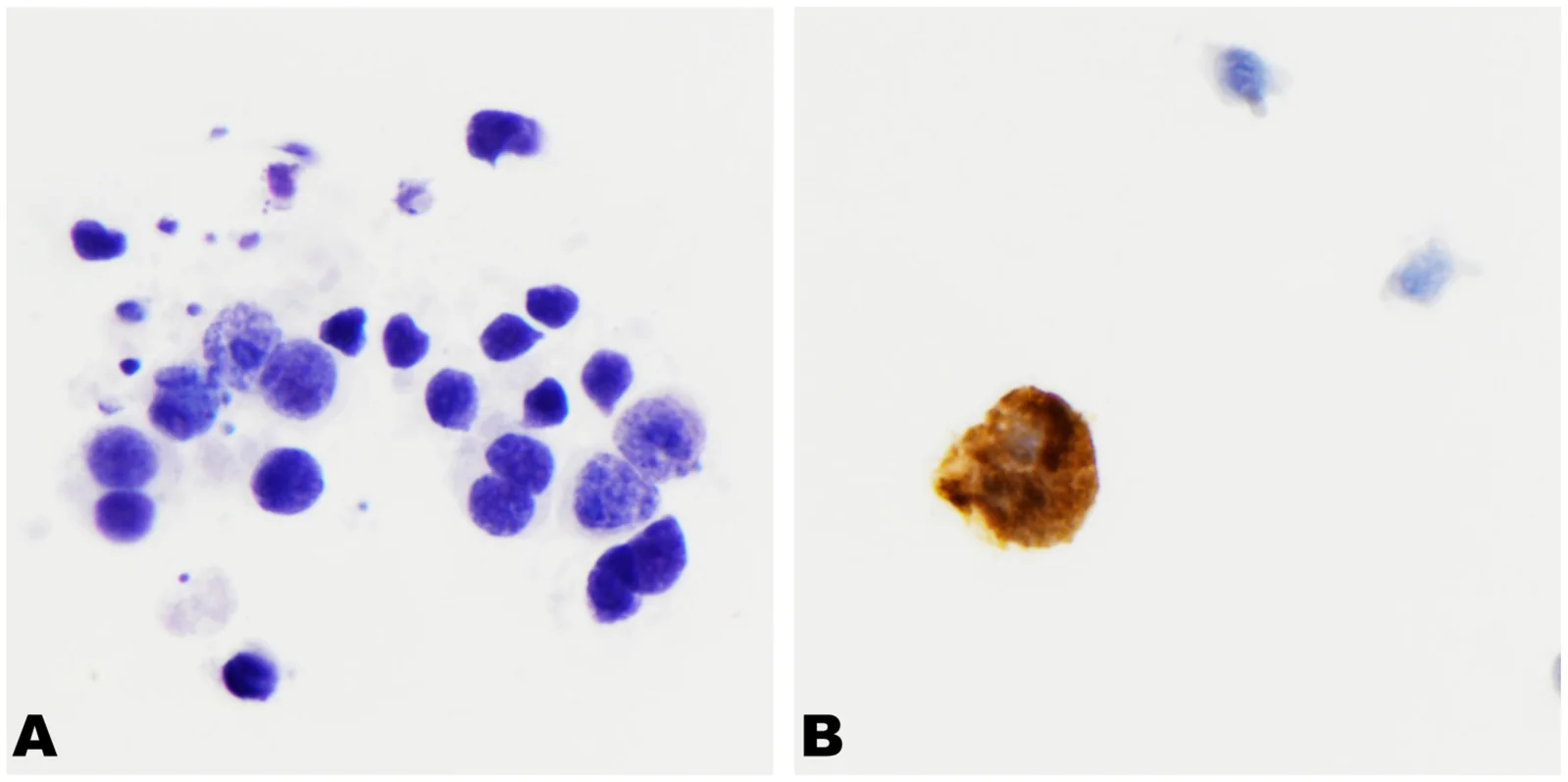
Cytological and immunocytochemical features of vitreoretinal lymphoma. (**A**) Papanicolaou-stained vitreous specimen showing large atypical lymphoid cells with high nuclear-to-cytoplasmic ratio, irregular nuclear contours and prominent nucleoli. (**B**) CD20 immunostaining demonstrating membranous positivity in an atypical lymphoid cell, supporting B-cell lineage. Original magnification: 400×. Original image from MIOS SA, Lausanne, Switzerland.

Cytology directly identifies VRL by demonstrating malignant cells. Lymphomatous cells are typically two to four times larger than normal lymphocytes and show a high nuclear-to-cytoplasmic ratio, irregular nuclear contours, coarse chromatin and prominent nucleoli. The cytoplasm is usually scant, although better-preserved cells may appear more basophilic [2,56,64] (Figure 6). Reactive lymphocytes, macrophages and cellular debris may also be present in the specimen [57,65].

The diagnostic performance of cytology varies considerably. Across 19 retrospective studies, lymphomatous cells were identified in 368/477 VRL eyes, corresponding to an aggregated sensitivity of 77.2%. Interpretation across studies is complicated by heterogeneous definitions of cytological positivity: some studies included suspicious or atypical cells, whereas others required unequivocal malignant cells or Papanicolaou class V cytology [8].

Immunocytochemical analysis can complement cytological morphology by establishing the lineage of atypical cells. CD20 is the most used B-cell marker, with CD79a and PAX5 providing additional confirmation, whereas CD3 identifies T cells [2,56,65]. CD45 may confirm haematolymphoid origin, while CD68 or CD163 can help distinguish macrophages from large atypical lymphoid cells in specimens with a prominent histiocytic component. Previous rituximab exposure is an important pitfall because CD20 expression may be markedly reduced or absent; CD79a or PAX5 may therefore be more informative in previously treated patients [56].

When cell-block material is available, immunohistochemistry allows broader phenotypic characterisation. In the cell-block series by Ohe et al., CD20 and CD79a were expressed in 100% of evaluable specimens, whereas MUM1 and BCL6 were positive in 85.7% and 57.1%, respectively; the mean Ki-67 labelling index was 66.3 ± 12.7% [62]. These markers provide additional phenotypic information but are not required to establish the diagnosis.

In the Santos et al. series of 237 vitreous biopsies, cytological smears showed 83% sensitivity and 98% specificity, with a diagnostic yield of 90%. In the same cohort, immunohistochemistry showed 92% sensitivity and 98% specificity, while the combination of cytological smears and immunohistochemistry reached 93% sensitivity. After immunohistochemistry, cytokine analysis, IGH rearrangement testing and flow cytometry were incorporated into the diagnostic work-up, and cytological smear sensitivity increased from 73% to 87%. The authors suggested that simultaneous interpretation of cytology and immunohistochemistry may have contributed to this improvement [56].

Flow cytometry characterises the lymphoid population using B-cell markers such as CD19 and CD20 together with κ and λ light chains [2,64,65]. Light-chain restriction supports B-cell clonality but is not proof of malignancy and should be interpreted with morphology and the clinical context. Because surface immunoglobulin expression may be absent in some DLBCLs, failure to demonstrate light-chain restriction does not exclude VRL [65].

In the Santos et al. series, flow cytometry was attempted in 103 biopsies but yielded an interpretable result in only 51, corresponding to a diagnostic yield of 50%. Among the 51 interpretable specimens, sensitivity and specificity were both 100%; however, these estimates exclude the 52 uninterpretable samples and were based on very few lymphoma-positive specimens [56]. The diagnostic utility of flow cytometry may be limited in paucicellular vitreous samples [57]. Its value may be greater when cellular morphology is degraded. Gascon et al. reported three samples in which cytology was inconclusive and flow cytometry identified a lymphomatous population in two, including one specimen containing only ten monoclonal B cells. Transport time was significantly longer in specimens showing cytological damage than in those without evident degeneration (116 vs. 79 min; *p* = 0.03), whereas flow-cytometric interpretation was less affected by transport time. In the same series, sensitivity was 81.8% for interpretable cytology and 91.7% for flow cytometry, with 100% specificity for both, although the cohort comprised only 19 patients [64].

Across six retrospective studies, flow cytometry was positive in 66/75 PVRL eyes, corresponding to an aggregated sensitivity of 88.0%, although study methods and the handling of insufficient or repeated samples varied considerably [8].

## 8. Cytokine Analysis

Cytokine measurements have been investigated for two distinct clinical purposes: supporting the initial diagnosis of VRL and monitoring disease activity after treatment. These applications should be considered separately because they address different clinical questions and may rely on different thresholds and sampling conditions.

### 8.1. Diagnostic Cytokine Biomarkers

Cytokine analysis provides diagnostic information in suspected VRL independently of lymphomatous cell preservation. Assessment is based on intraocular IL-10 concentration and its relationship to interleukin-6 (IL-6). IL-10 is produced by normal and neoplastic B-cells, while IL-6 is associated with intraocular inflammation; the IL-10/IL-6 ratio therefore helps distinguish a lymphoma-predominant cytokine profile from a reactive one [2,8,66]. An IL-10/IL-6 ratio > 1 is used as supportive evidence of B-cell VRL, particularly in vitreous samples, and is included in current consensus recommendations [2]. The threshold varies according to the ocular compartment: while a ratio > 1 is generally applied to vitreous samples, a cut-off of 0.6 has been evaluated in aqueous humour [66].

Absolute IL-10 cut-offs also vary between studies. Cassoux et al. reported thresholds of 50 pg/mL in aqueous humour and 400 pg/mL in vitreous, with sensitivities of 89% and 80% and specificities of 93% and 99%, respectively [67]. Liu et al. applied lower thresholds of >30 pg/mL and >65 pg/mL in aqueous and vitreous humour, respectively [68]. Differences in assay methodology and the lack of international standardisation of cytokine measurements account for this variability [8,66].

Nevertheless, pooled data indicate a high diagnostic performance. Huang et al. analysed 29 retrospective studies comprising 662 eyes with VRL and found an aggregated sensitivity of 89.4% (278/311 eyes) for an IL-10/IL-6 ratio > 1. In subgroup analysis, sensitivity was 88.9% in vitreous samples and 83.3% in aqueous samples, with no significant difference between compartments (*p* = 0.42). Because the review included only PVRL cohorts, specificity could not be assessed [8].

A meta-analysis included 41 studies and 2411 patients with VRL or ocular inflammatory disease. Across 27 studies evaluating aqueous and/or vitreous samples, the IL-10/IL-6 ratio showed a pooled sensitivity of 0.89 and specificity of 0.98, with an area under the summary receiver operating characteristic curve (AUSROC) of 0.98. Subgroup analysis by ocular compartment showed a sensitivity of 0.95 and specificity of 1.00 in aqueous humour, compared with 0.86 and 0.97 in vitreous samples, respectively, although these estimates were derived from different study subsets. IL-10 alone showed a pooled sensitivity of 0.87 and specificity of 0.95 across 11 studies using different cut-off values; subgroup analysis suggested better diagnostic performance with thresholds <100 pg/mL. There was no significant difference in overall diagnostic accuracy compared with the IL-10/IL-6 ratio. The certainty of evidence was low because most studies were retrospective, cut-off values varied and between-study heterogeneity was substantial [69].

The compartment-specific approach is supported by studies directly comparing aqueous and vitreous cytokine concentrations. Liu et al. analysed paired aqueous and vitreous samples from 74 eyes of 64 patients with biopsy-proven B-cell VRL. Median IL-10 concentration was substantially higher in vitreous than in aqueous humour (1159.77 vs. 225.74 pg/mL; *p* < 0.001). An IL-10/IL-6 ratio >1 was positive in 89% of vitreous samples and 77% of aqueous samples [68].

These differences were also related to the anatomical distribution of lymphoma. In the same cohort, intraretinal infiltration was independently associated with higher IL-10 concentrations in both aqueous and vitreous humour, whereas vitreous haze and RPE involvement were not. Aqueous IL-10 >30 pg/mL was positive in 97% of eyes with intraretinal infiltration compared with 78% of eyes without it [68]. Thus, a negative aqueous IL-10 result should be interpreted cautiously, particularly in eyes without intraretinal infiltration.

Rather than relying on a single cut-off, the Interleukin Score for IntraOcular Lymphoma Diagnosis (ISOLD) combines IL-10 and IL-6 concentrations in a probabilistic model, using separate equations for aqueous and vitreous samples. A score > 0 corresponds to a probability >50% of B-cell VRL [66].

The performance of ISOLD was also assessed by Chevalier et al. in 183 samples from 166 patients, including 25 patients with confirmed VRL; 156 samples were aqueous and 27 were vitreous. At the established ISOLD threshold of 0, aqueous samples showed 77.8% sensitivity, 98.6% specificity, 87.5% positive predictive value and 97.1% negative predictive value. Corresponding values for vitreous samples were 95%, 100%, 100% and 87.5%, respectively, although the small vitreous subgroup limits the precision of these estimates. ROC analysis identified lower cohort-specific optimal thresholds for both ISOLD and the IL-10/IL-6 ratio; however, these values were reported as descriptive optima for this study population and were not proposed as replacements for the established ISOLD threshold of 0 or its original probability-based interpretation [66].

The same study also illustrates why cytokine assays cannot establish VRL in isolation. Two false-positive ISOLD results occurred in varicella-zoster virus-associated ocular inflammation, whereas five VRL samples were falsely negative; two of the latter were obtained from patients receiving systemic corticosteroids and/or chemotherapy [66]. Other reports have also described increased intraocular IL-10 in viral retinal necrosis and ocular toxoplasmosis [8,66]. Conversely, low cytokine concentrations may occur during treatment, in immunosuppressed patients or when the anatomical distribution of lymphoma results in limited release into the sampled compartment [66,68]. ISOLD was developed for B-cell intraocular lymphoma and should not be applied to T-cell or NK-cell VRL [66].

### 8.2. Biomarkers for Treatment Monitoring and Recurrence

Serial cytokine measurements have also been investigated as markers of treatment response and ocular recurrence rather than as tests for initial diagnosis. Park et al. followed 14 patients with VRL treated with intravitreal methotrexate and serially measured aqueous cytokines. IL-10 and the IL-10/IL-6 ratio decreased significantly one month after treatment initiation; the mean ratio fell from 141.23 ± 244.71 to 0.98 ± 1.45 (*p* = 0.001). Mean time to IL-10 normalisation was 1.17 ± 0.40 months, requiring a mean of 8.54 ± 1.92 methotrexate injections. Six patients experienced disease recurrence, and aqueous IL-10 increased again at recurrence and decreased after retreatment in all six [70].

Similar findings were reported by Gu et al. in 58 eyes of 33 patients receiving intravitreal methotrexate. Aqueous IL-10 followed the course of treatment and was more accurate than the IL-10/IL-6 ratio for detecting ocular relapse. An IL-10 threshold >50 pg/mL yielded 100% sensitivity and 95.1% specificity, with an AUSROC of 0.992; by comparison, an IL-10/IL-6 ratio > 1 showed 61.5% sensitivity, 87.8% specificity and an AUSROC of 0.921 [71].

## 9. Molecular Diagnostics

Molecular testing can support the diagnosis of suspected VRL [2,65]. Current methods include PCR-based assessment of IGH gene rearrangements, mutation-specific testing, mainly MYD88 L265P and CD79B, and targeted next-generation sequencing (NGS) [1,2,65].

PCR analysis of IGH gene rearrangements is used to detect a dominant B-cell clone. Lou et al. analysed 12 studies comprising 649 patients and reported a pooled sensitivity of 0.85, specificity of 0.97 and AUSROC of 0.97 for IGH testing [69]. Detection of a clonal IGH rearrangement supports B-cell clonality but does not establish malignancy. Extensive somatic hypermutation may interfere with primer binding and produce false-negative results, whereas low cellularity can generate pseudoclonal patterns. Clonal or oligoclonal B-cell expansions may also occur in ocular inflammatory conditions [3,65]. Clonality results therefore require correlation with cytology, immunophenotype and the clinical context. The analytical strategy is also relevant: multitarget IGH approaches covering different framework regions showed higher sensitivity than single-target strategies (0.88 vs. 0.72) and a higher diagnostic odds ratio, although the subgroup difference did not reach statistical significance [69].

The most widely used mutation-specific assay targets MYD88 L265P. Across 12 studies and 382 patients, Lou et al. reported a pooled sensitivity of 0.73, specificity of 0.99 and AUSROC of 0.89. CD79B mutation testing showed lower sensitivity (0.40) but a specificity of 1.00 across four studies and 123 patients [69]. The low sensitivity of CD79B limits its value as an initial marker, although a positive result may provide additional molecular support. A negative MYD88 result therefore does not exclude VRL. Conversely, detection of MYD88 L265P does not establish a primary ocular origin, as the mutation may also occur in lymphomas with secondary vitreoretinal involvement [8]. MYD88 L265P is primarily a marker of B-cell malignancy and should not be relied upon to detect T-cell or NK-cell subtypes [8,65].

Analytical sensitivity depends on the method used. Conventional sequencing may fail when the mutant allele fraction is low, whereas allele-specific PCR and droplet digital PCR (ddPCR) allow for detection of lower mutant allele fractions [65]. In paired pretreatment samples, Hiemcke-Jiwa et al. evaluated MYD88 L265P by ddPCR in ocular fluid and reported 75% sensitivity and 100% specificity in vitreous samples, compared with 67% sensitivity and 100% specificity in aqueous humour [12]. These results show that aqueous humour can be used for MYD88 testing, although a negative aqueous result cannot exclude VRL. In the meta-analysis by Lou et al., MYD88 diagnostic performance did not differ significantly by sample type. Aqueous samples showed slightly higher sensitivity than vitreous samples (0.77 vs. 0.70), whereas vitreous samples showed numerically higher specificity (0.99 vs. 0.94) and diagnostic odds ratio [69]. Thus, aqueous humour allows mutation testing from a small, minimally invasive sample, whereas vitreous is preferable when both cellular and molecular analyses are required.

Targeted NGS allows multiple genomic alterations to be assessed simultaneously from a small ocular sample. Cani et al. applied a 126-gene panel to diluted vitreous samples from four patients with VRL and obtained sufficient DNA for sequencing in all cases. The use of diluted vitreous preserved undiluted material for cytology and flow cytometry; in one case, NGS detected tumour DNA in a banked vitreous specimen that had originally been cytologically negative [72]. NGS can identify multiple VRL-associated alterations when single-gene testing is negative. However, current evidence is based mainly on small cohorts and performance depends on panel design, sequencing depth, DNA quality and bioinformatic thresholds [65,72].

Parallel testing of the IL-10/IL-6 ratio and MYD88 L265P, with either test considered positive, achieved 97.1% sensitivity and 97.0% specificity and reduced the false-negative rate from 11% to 2.9% [69].

## 10. Towards Precision Diagnosis: An Integrated Diagnostic Framework

**Figure 7:**
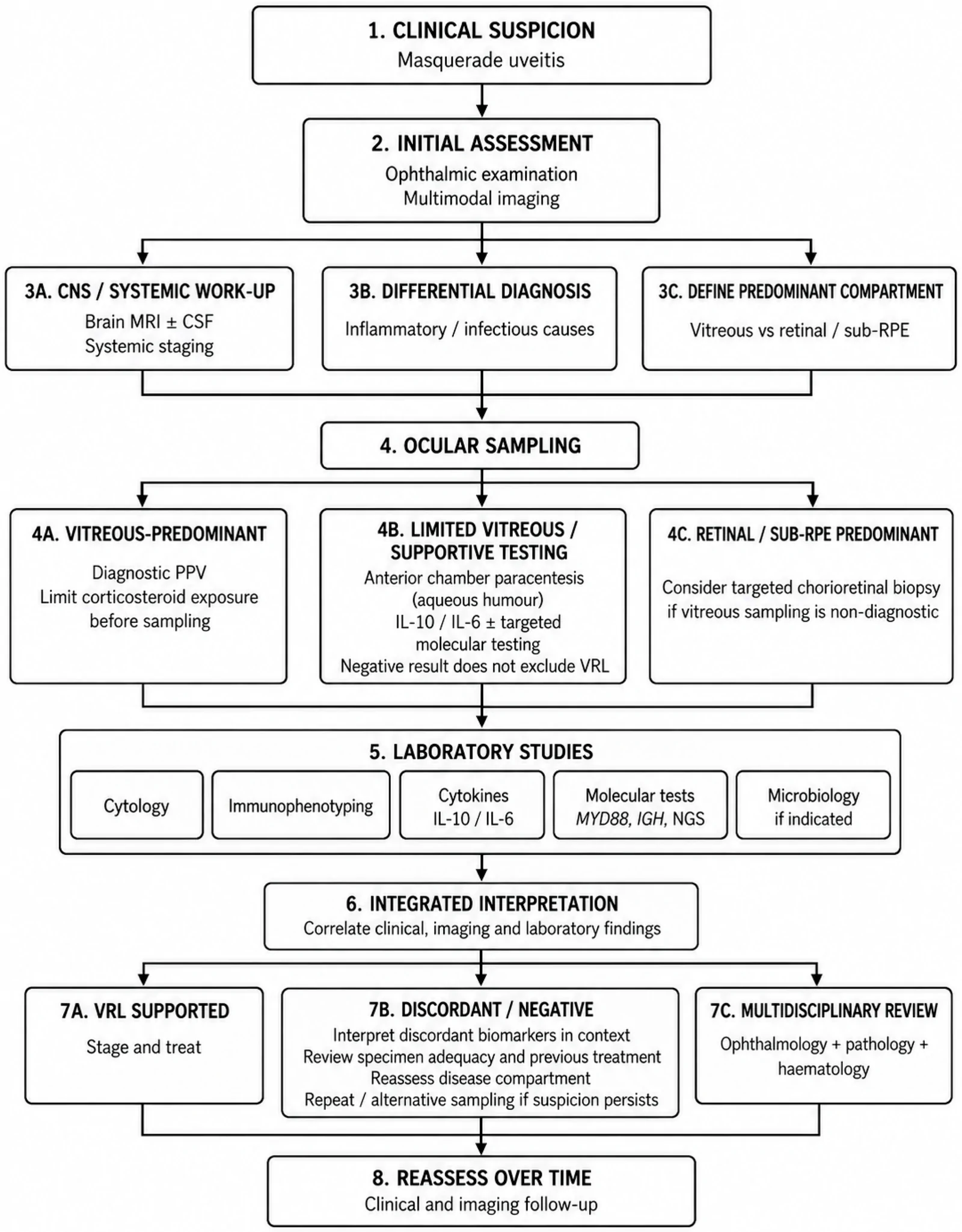
Proposed diagnostic workflow for suspected vitreoretinal lymphoma. The algorithm integrates clinical suspicion, multimodal imaging, CNS/systemic assessment, identification of the predominant ocular compartment and tailored ocular sampling. In eyes with limited vitreous involvement, aqueous humour obtained by anterior chamber paracentesis may be used for cytokine and targeted molecular testing. Cytology, immunophenotyping, cytokine analysis and molecular testing are interpreted together with clinical and imaging findings to support diagnosis and guide repeat or alternative sampling when results are inconclusive.

Diagnosis of VRL relies on the combined interpretation of clinical, imaging and laboratory findings, with the diagnostic approach guided by the predominant anatomical site and the ocular specimen available for analysis [1,2,9]. CNS and systemic evaluation should proceed in parallel to identify associated or secondary lymphoma [1,2,3]. When results are negative or discordant, specimen adequacy, previous treatment and disease distribution should be reconsidered; persistent clinical suspicion may warrant repeat sampling or biopsy of an alternative site [2]. Discordant results should be interpreted according to the diagnostic role and limitations of each test. Positive cytology provides direct evidence of lymphoma even when MYD88 L265P is not detected, as a negative MYD88 result does not exclude VRL [2,56,65,69]. Conversely, elevated IL-10 with negative MYD88 provides supportive but not confirmatory evidence and should be interpreted together with cytology, imaging and the exclusion of infectious or inflammatory mimics [2,66,69]. Negative cytology does not exclude VRL when the specimen is paucicellular, degraded or obtained from a compartment with limited tumour involvement. If cytology, cytokine and molecular studies are all negative but imaging remains strongly suggestive, specimen adequacy, previous corticosteroid exposure and the anatomical distribution of disease should be reassessed; persistent suspicion may warrant repeat vitrectomy or targeted chorioretinal biopsy [2]. Formal cost-effectiveness comparisons between diagnostic strategies are lacking. From a practical standpoint, when sufficient vitreous material is available, parallel allocation to cytology and immunophenotyping, IL-10/IL-6 assessment and *MYD88* testing provide complementary cellular, soluble and molecular information [2,56,65,69]. *IGH* clonality testing or broader NGS may be reserved for unresolved or discordant cases, particularly when cellular material is limited. The proposed diagnostic framework is shown in Figure 7.

## 11. Emerging Diagnostic Strategies

Emerging diagnostic approaches include image-based machine learning, liquid biopsy, single-cell genomics and high-dimensional molecular profiling [72,73,74,75,76,77,78,79,80,81].

Khan et al. developed a deep-learning model to distinguish VRL from non-infectious uveitis using conventional posterior-segment OCT B-scans. Individual B-scans were analysed by a convolutional neural network and combined to obtain an eye-level classification. Grad-CAM heatmaps identified the image regions contributing most to the prediction, highlighting preretinal deposits and RPE abnormalities in VRL, whereas vitreous opacities, epiretinal membranes, intraretinal cells and choroidal or scleral abnormalities were more associated with uveitis. The model achieved a mean accuracy of 75.4%, sensitivity of 77.7%, specificity of 73.4% and AUSROC of 0.76 across five-fold cross-validation [73].

Gozzi et al. applied radiomic analysis to anterior vitreous images acquired with anterior-segment OCT to distinguish VRL from inflammatory vitritis. After manual segmentation of the anterior vitreous, quantitative texture features were extracted from each image and used to train an XGBoost classifier. The best-performing model, based on 128 grey levels, achieved a test AUSROC of 0.84 and correctly classified 41/47 eyes (87%) and 23/28 patients (82%) [74].

Li et al. developed a non-invasive machine-learning model for PVRL screening based on six routinely blood count parameters: platelet distribution width, monocyte percentage, platelet large cell ratio, absolute monocyte count, haemoglobin and absolute basophil count. These variables were combined in a random-forest classifier that achieved an AUSROC of 0.85 in the discovery cohort and 0.80–0.83 across independent validation cohorts. The model showed 95.0% sensitivity, 99.97% specificity, a positive predictive value of 57.6% and a negative predictive value of 99.99%. However, not all individuals classified as low-risk underwent biopsy confirmation, and low-risk participants in the community-based cohort were not systematically followed [75].

Molecular approaches that reduce dependence on intact lymphomatous cells are also being explored. Cell-free DNA (cfDNA) analysis enables molecular profiling of extracellular DNA fragments released into intraocular fluid, retaining diagnostic information even when malignant cells are scarce. Gu et al. analysed vitreous samples from 23 eyes with VRL and 25 eyes with inflammatory eye disease using both cytopathology and cfDNA sequencing. cfDNA sequencing was successfully completed in all VRL samples and in 20 of 25 inflammatory controls. A diagnostic model based on MYD88 and ETV6 mutations achieved 91.3% sensitivity and 95% specificity, compared with 82.6% and 100%, respectively, for cytopathology [76].

Single-cell genomic analysis can extract molecular information from individual B cells recovered from paucicellular vitreous samples. Tan et al. examined specimens from seven patients with VRL and four with chronic inflammation using DEPArray-based B-cell isolation. VRL samples showed higher frequencies of the dominant IGH clonotype (88.8% ± 13.2% vs. 65.9% ± 13.4%) and homozygous MYD88 L265P mutations (35.0% ± 31.3% vs. 1.5% ± 2.6%). In one cytology-confirmed case, 11 of 15 B cells shared identical paired IGH:IGK rearrangements, while MYD88 L265P and BCL2/JH t(14;18) were each detected in 14 of 15 cells [77].

Ocular-fluid microRNAs have also been investigated. Tuo et al. prospectively analysed samples from 17 patients with B-cell VRL and 12 with uveitis. Among candidate microRNAs identified using an initial 168-miRNA panel, only miR-155 remained significantly different on subsequent individual analysis, with higher levels in uveitis than in VRL. Substantial overlap between groups limited its discriminatory value [78].

Funatsu et al. performed high-dimensional proteomic profiling of vitreous samples from 11 VRL eyes and four macular-hole control eyes using the aptamer-based SomaScan 7K platform. Among 6981 quantified proteins, 2499 met predefined differential-expression criteria, with 2440 upregulated and 59 downregulated in VRL. Prominently upregulated proteins included histone H3 and H4 family members, runt-related transcription factor 1 (RUNX1), histone deacetylase 1 (HDAC1) and protein arginine methyltransferase 1 (PRMT1). Pathway analysis showed enrichment of nuclear and chromatin-related processes, including DNA methylation, PRC2-mediated histone modification and histone deacetylation, whereas proteins associated with extracellular-matrix organisation, vesicle and secretory components, and growth-factor signalling were relatively reduced. Unsupervised analysis clearly separated VRL from control samples [79].

These approaches differ substantially in their proximity to clinical implementation. OCT-based deep learning and blood-count models use routinely acquired clinical data [73,75], but current performance estimates derive predominantly from relatively small or retrospectively assembled cohorts and require external validation across centres, populations and imaging devices. Radiomic approaches requiring manual segmentation may be less readily scalable [74], whereas cfDNA sequencing and single-cell analyses provide more extensive molecular information but require specialised laboratory infrastructure [76,77]. At present, these methods should be considered adjuncts to the diagnostic workflow rather than replacements for established cellular, cytokine or molecular testing.

Unlike the preceding diagnostic studies, longitudinal molecular profiling has primarily been explored as an approach to treatment monitoring and biological response rather than as a test for initial diagnosis. Our group reported the first vitreous detection and longitudinal assessment of IL-16 during intravitreal methotrexate/dexamethasone treatment [80]. IL-10 became undetectable by day 22, whereas IL-16 declined more slowly and remained detectable until day 48, indicating different treatment-related kinetics between lymphoma-associated cytokine production and the broader intraocular immune microenvironment. The same patient was profiled using a 58-mediator panel across nine serial 0.2 mL vitreous samples. CXCL12 and CXCL13 declined more slowly than IL-10, while CCL2 remained elevated and HGF persistently high. These differing kinetics may reflect a dynamic interplay between the tumour and the host immune response, while the serial analysis demonstrates the feasibility of longitudinal multiplex profiling from very small vitreous samples [81]. These observations remain exploratory because they derive from a single patient receiving concomitant intravitreal methotrexate and dexamethasone and do not establish diagnostic or recurrence thresholds [80,81].

## 12. Gaps, Controversies and Limitations

Several sources of heterogeneity limit direct comparison of diagnostic studies in VRL. Reported cytological sensitivity varies widely because studies differ in specimen volume and cellularity, previous corticosteroid exposure, sampling and processing protocols, definitions of cytological positivity, pathology expertise and the handling of insufficient or repeated specimens [2,8,56,57,58,59,60,61,62,63,64]. Cytokine thresholds are similarly influenced by the ocular compartment sampled, assay methodology, sample dilution, treatment status, case mix and the method used to derive the diagnostic cut-off [8,66,67,68,69]. Consequently, pooled diagnostic estimates should not be interpreted as universally applicable thresholds.

Most available evidence derives from retrospective, single-centre or relatively small multicentre cohorts with heterogeneous reference standards. Emerging molecular and computational approaches are additionally limited by variable analytical platforms and limited independent external validation [73,74,75,76,77,78,79]. Studies evaluating treatment response or recurrence should also be distinguished from diagnostic studies because longitudinal biomarker kinetics address a different clinical question [70,71,80,81]. Prospective multicentre studies using standardised specimen handling, prespecified diagnostic thresholds and independent validation are needed to determine the incremental value of individual tests and test combinations.

## 13. Conclusions

VRL remains difficult to diagnose because no single test is sufficiently sensitive across all clinical settings. Cytology provides direct evidence of lymphoma but depends on cellular yield and preservation, whereas cytokine and molecular assays can remain informative in paucicellular samples. Multimodal imaging defines the anatomical distribution of disease and helps identify the ocular compartment most likely to yield diagnostic material. Diagnostic biomarkers should be distinguished from markers used longitudinally to assess treatment response or recurrence. In practice, the most reliable approach is the specimen- and compartment-specific integration of morphological, imaging, cytokine and molecular findings, with repeat or alternative sampling when results remain discordant. Timely diagnostic clarification also permits appropriate CNS staging and disease-specific management. Emerging AI-based imaging, and radiomic and molecular approaches remain investigational and require prospective external validation before incorporation into routine diagnostic pathways.

## Figures and Tables

**Table 1:** Clinical findings increasing or decreasing suspicion of vitreoretinal lymphoma, synthesised from consensus recommendations and clinical series [1,2,3,4,36,37,38,39].

Clinical Domain	Findings Increasing Suspicion of VRL	Findings Decreasing Suspicion of VRL
Demographic profile	Older adult, most commonly ≥ 50 years	—
Symptoms	Floaters, blurred or hazy vision, painless visual loss	Ocular pain, redness, photophobia
Laterality	Frequently bilateral and asymmetric	—
Anterior segment	Mild anterior chamber cells; keratic precipitates of variable morphology	Scleritis, pseudohypopyon, anterior or posterior synechiae
Vitreous	Cellular infiltration; sheets, veils, strands or clumps; “aurora borealis” appearance	Snowballs, snowbanking, vitreous haemorrhage
Retina/RPE	Multifocal creamy/white outer-retinal or RPE infiltrates; deep white retinal dots; “leopard-skin” pigmentation; RPE atrophy or fibrosis	Retinal holes; marked retinal haemorrhage
Inflammatory phenotype	Dense vitreous infiltration with relatively little macular oedema	Prominent macular oedema; marked retinal vasculitis
Response to corticosteroids	Partial or transient improvement followed by recurrence or progression, particularly during dose reduction	Sustained response to corticosteroid monotherapy consistent with immune-mediated uveitis

**Table 2:** Multimodal imaging findings in vitreoretinal lymphoma: synthesis of reported features, quantitative evidence and clinical limitations [41,42,43,44,45,46,47,48,49,50,51,52,53,54,55].

Imaging Modality	Characteristic Features	Main Quantitative Evidence	Clinical Relevance and Limitations
Colour fundus photography/UWF imaging	Vitreous haze and cellular aggregates; retinal or subretinal lesions; RPE mottling; perivascular sheathing	UWF imaging disclosed additional abnormalities beyond the conventional 30° field in 47% of eyes [41]	Defines lesion distribution and may reveal peripheral involvement not captured by conventional fundus photography; findings are not diagnostic
FAF	Granular or stippled pattern of mixed hyper- and hypoautofluorescent foci; RPE atrophy following lesion regression	Mixed hypo-/hyperautofluorescent pattern in 65.99% of eyes; normal FAF in 32.17% [42]	Useful for assessment of RPE abnormalities and longitudinal imaging; FAF alone does not reliably distinguish active from inactive disease, and normal FAF does not exclude VRL
Colour-coded FAF	Granular pattern; retinitis-like lesions; lymphomatous RPE detachments; sub-RPE deposits; RPE atrophy; perivascular infiltrates	Retinitis-like lesions detected in 2/2 eyes versus 0/2 with blue or green FAF; lymphomatous RPE detachments in 4/4 versus 1/4 with either conventional modality [43]	Complementary technique that may improve qualitative delineation of selected lesions; evidence is limited to a small retrospective series
Structural OCT	Sub-RPE, subretinal and intraretinal hyper-reflective infiltration; PED; fuzzy outer-retinal changes; vertical hyper-reflective lesions; preretinal deposits; RPE disruption	Sub-RPE involvement in 91%, subretinal involvement in 43% and intraretinal involvement in 7% of 182 eyes [45]	Provides high-resolution anatomical localization and longitudinal structural assessment; no single OCT feature is diagnostic
En face OCTA	Perivascular flower-bud-like lesions appearing as punctate points or confluent bands surrounding retinal vessels on midretinal slabs	PFBLs detected in 12/35 eyes (34.3%); 10/12 were identified within 6 months of symptom onset [51]	May reveal early perivascular intraretinal abnormalities; PFBLs are not always detectable on fundus photography or FA, and evidence is currently based on a single retrospective cohort
Retromode imaging	Pseudo-three-dimensional visualisation of deep-retinal and RPE abnormalities	Seven eyes from five patients were analysed; additional lesions compared with FAF were identified in all analysed eyes [52]	May disclose abnormalities outside routine macular imaging and guide targeted OCT acquisition; image quality is strongly affected by vitritis and other media opacities
Quantitative SS-OCT/OCTA of the choroid	Reduced CVI; altered choriocapillaris perfusion; treatment-related changes in CT, CVV and CSV	Evaluated in 44 eyes from 29 patients; baseline CVI was significantly lower than in controls across all analysed ETDRS regions [53]	Exploratory quantitative assessment of choroidal changes and treatment response; findings are not validated diagnostic biomarkers and may partly reflect reactive inflammatory-cell infiltration
FA	Mottled or “leopard-spot” pattern; round hypofluorescent spots; window defects; staining lesions; optic disc and vascular leakage	Leopard spots 51.2%; multiple hypofluorescent spots 40.6%; window defects 40.8%; staining lesions 33.9% [42]	Provides complementary information on RPE-associated and vascular abnormalities; inflammatory angiographic findings may coexist and are not specific for VRL
ICGA	Small hypocyanescent spots, usually fewer than corresponding hypofluorescent lesions on FA and often becoming less conspicuous in late phases	Vanishing hypocyanescent spots 40.7%; persistent hypocyanescent spots 7.5% [42]	Complementary role; available quantitative estimates are based on only two studies
B-scan ultrasonography	Retinal thickening or occupying lesions; centrifugal condensation of vitreous haze, characterised by peripheral hyperreflectivity adjacent to the posterior vitreous cortex with relative central hyporeflectivity; vitreoretinal adhesion is less frequent	In 106 eyes with biopsy-confirmed VRL and 86 eyes with uveitis, retinal thickening or occupying lesions were observed in 20% vs. 1% (OR 19.07), centrifugal condensation in 46% vs. 15% (OR 4.83), and vitreoretinal adhesion in 6% vs. 23% (OR 0.20) [55]	May provide diagnostic clues when media opacity limits fundus imaging and facilitate selection of patients for further investigation; findings are not specific, and interpretation is affected by operator dependence and ultrasound gain settings

## Data Availability

No new datasets were generated or analysed in this study. Data sharing is not applicable to this article.
